# Hyperspectral image processing for the identification and quantification of lentiviral particles in fluid samples

**DOI:** 10.1038/s41598-021-95756-3

**Published:** 2021-08-10

**Authors:** Emilio Gomez-Gonzalez, Beatriz Fernandez-Muñoz, Alejandro Barriga-Rivera, Jose Manuel Navas-Garcia, Isabel Fernandez-Lizaranzu, Francisco Javier Munoz-Gonzalez, Ruben Parrilla-Giraldez, Desiree Requena-Lancharro, Manuel Guerrero-Claro, Pedro Gil-Gamboa, Cristina Rosell-Valle, Carmen Gomez-Gonzalez, Maria Jose Mayorga-Buiza, Maria Martin-Lopez, Olga Muñoz, Juan Carlos Gomez Martin, Maria Isabel Relimpio Lopez, Jesus Aceituno-Castro, Manuel A. Perales-Esteve, Antonio Puppo-Moreno, Francisco Jose Garcia Cozar, Lucia Olvera-Collantes, Silvia de los Santos-Trigo, Emilia Gomez, Rosario Sanchez Pernaute, Javier Padillo-Ruiz, Javier Marquez-Rivas

**Affiliations:** 1 Department of Applied Physics III, School of Engineering, Universidad de Sevilla, Camino de los Descubrimientos s/n, 41092 Sevilla, Spain; 2 Institute of Biomedicine of Seville, 41013 Sevilla, Spain; 3 Unidad de Producción y Reprogramación Celular (UPRC), Red Andaluza de Diseño y Traslación de Terapias Avanzadas, 41092 Sevilla, Spain; 4 School of Biomedical Engineering, The University of Sydney, Sydney, NSW 2006 Australia; 5 EOD-CBRN Group, Spanish National Police, 41011 Sevilla, Spain; 6 Technology and Innovation Centre, Universidad de Sevilla, 41012 Sevilla, Spain; 7 Service of Intensive Care, University Hospital ‘Virgen del Rocio’, 41013 Sevilla, Spain; 8 Service of Anaesthesiology, University Hospital ‘Virgen del Rocio’, 41013 Sevilla, Spain; 9 Instituto de Astrofísica de Andalucía, CSIC, 18008 Granada, Spain; 10 Department of Ophthalmology, University Hospital ‘Virgen Macarena’, 41009 Sevilla, Spain; 11 OftaRed, Institute of Health ‘Carlos III’, 28029 Madrid, Spain; 12 Centro Astronomico Hispano Alemán, 04550 Almeria, Spain; 13 Department of Electronic Engineering, School of Engineering, Universidad de Sevilla, 41092 Sevilla, Spain; 14 Department of Biomedicine, Biotechnology and Public Health, University of Cadiz, 11003 Cadiz, Spain; 15 Instituto de Investigación E Innovación Biomedica de Cádiz (INIBICA), 11009 Cadiz, Spain; 16 Corporación Tecnológica de Andalucía, 41092 Sevilla, Spain; 17 Joint Research Centre, European Commission, 41092 Sevilla, Spain; 18 Department of General Surgery, University Hospital ‘Virgen del Rocío’, 41013 Sevilla, Spain; 19 Service of Neurosurgery, University Hospital ‘Virgen del Rocío’, 41013 Sevilla, Spain; 20 Centre for Advanced Neurology, 41013 Sevilla, Spain

**Keywords:** Near-infrared spectroscopy, Viral infection

## Abstract

Optical spectroscopic techniques have been commonly used to detect the presence of biofilm-forming pathogens (bacteria and fungi) in the agro-food industry. Recently, near-infrared (NIR) spectroscopy revealed that it is also possible to detect the presence of viruses in animal and vegetal tissues. Here we report a platform based on visible and NIR (VNIR) hyperspectral imaging for non-contact, reagent free detection and quantification of laboratory-engineered viral particles in fluid samples (liquid droplets and dry residue) using both partial least square-discriminant analysis and artificial feed-forward neural networks. The detection was successfully achieved in preparations of phosphate buffered solution and artificial saliva, with an equivalent pixel volume of 4 nL and lowest concentration of 800 TU·$\upmu$L^−1^. This method constitutes an innovative approach that could be potentially used at point of care for rapid mass screening of viral infectious diseases and monitoring of the SARS-CoV-2 pandemic.

## Introduction

A variety of spectroscopic methods have been used in many applications that range from the agro-food industry^1^ to biological sciences^2^, medical applications^3–5^, and the pharmacological industry^6^ among others. In particular, the use of near-infrared (NIR) spectroscopy has been reported for the detection of microorganisms in different media. For example, Yao-Zen and co-workers^7^ studied the use of NIR hyperspectral imaging to detect, in chicken fillets, the presence of *Enterobacteriaceae*, a large family of gram-negative bacteria able to cause important diseases. Similarly, Siripatrawan et al.^8^ reported the detection of *Escherichia coli* in packaged spinach. For this purpose, the authors implemented an artificial neural network (ANN) to successfully predict food contamination. While there is vast evidence that hyperspectral imaging can be used to detect relatively large microorganisms such as bacteria or fungi^9^, it is not clear whether these optical techniques can be successfully applied for the detection of viral particles. First, bacteria and fungi are approximately two orders of magnitude larger than viruses and tend to form colonies. Secondly, the size of viral particles typically lies slightly below the lower limit of optical wavelengths (< 400 nm). Despite the apparent limitations that may exist for the detection of viruses using optical spectroscopic imaging techniques, there are few studies that suggest its viability^10–13^. However, it is not clear whether these studies detected the presence of the virus or its effects on the tissues of the hosts, as the damage and tissue alterations caused during an on-going infection may appear more prominent than the virus itself at the working wavelengths.

**Figure 1:**
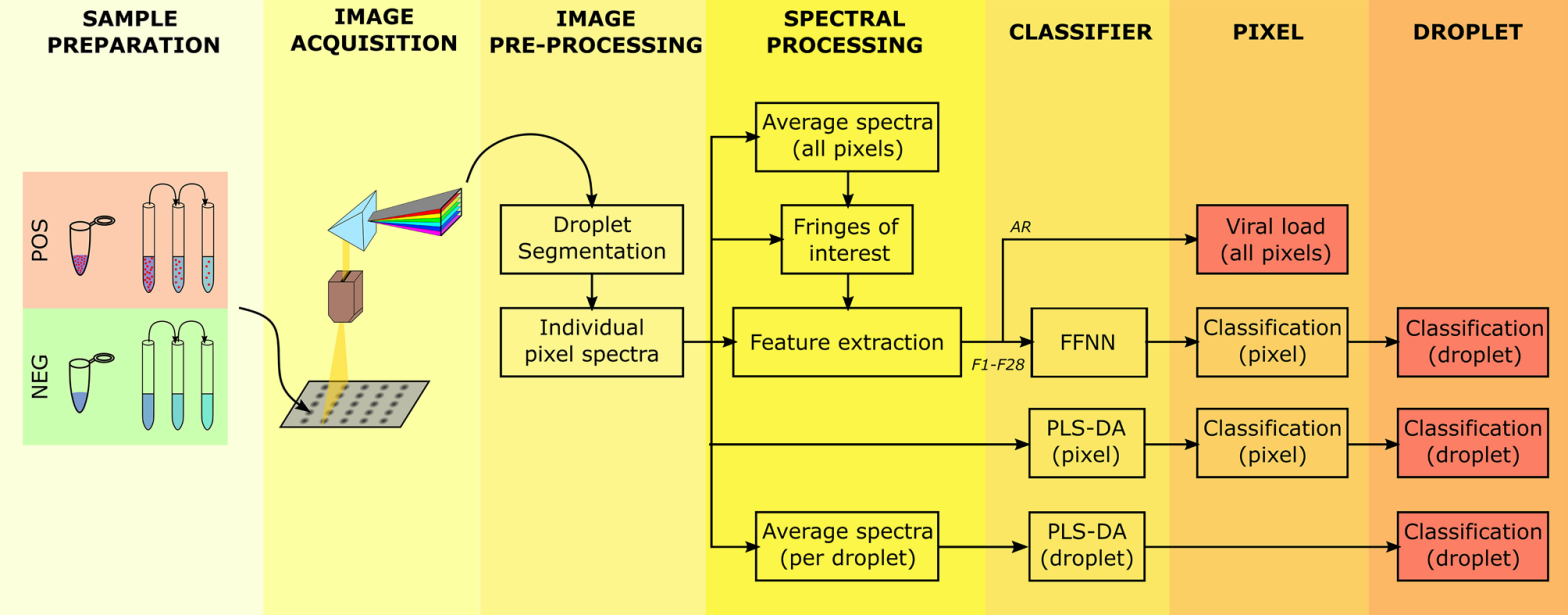
Block diagram of proposed processing platform for classification (positive or negative) of fluid samples with lentiviral particles and quantification of their viral load. Fluid samples are deposited on a supporting plate and imaged (as liquid droplets and dry residues) in the visible and near-infrared ranges using a hyperspectral camera. Optical diffuse reflectance spectra are obtained and processed from individual pixels and averaged. Spectral (wavelength) fringes of interest are determined for the calculation of 29 morphological descriptors of features of individual spectra (F1–F28) and the area ratio (AR) with respect to the spectrum of the background. Two independent classifiers are constructed and evaluated –using the same training and test sample groups– at the pixel and sample (droplet) levels: a partial least square discriminant analysis (PLS-DA) of individual and droplet-averaged spectra and an artificial feed-forward neural network (FFNN) built on the values of the descriptors of features of individual spectra.

Here, we pursued to understand whether visible and NIR (VNIR) hyperspectral imaging can be exploited to determine the presence of viral particles in a fluid suspension as well as on a surface upon complete evaporation of its water content. We analysed the diffuse optical reflectance spectra obtained from preparations of lentiviral particles pseudotyped with the glycoprotein G of the vesicular stomatitis virus (VSV) using three different approaches: classification (positive/negative) by partial least square-discriminant analysis (PLS-DA) and a feed-forward neural network (FFNN), and quantification of viral load by analysis of averaged spectra, as summarized in Fig. 1. The viral model under examination has been used to investigate a number of human diseases^14^ including the severe acute respiratory syndrome coronavirus 2 (SARS-CoV-2)^15^, as it provides a safer alternative while lowering biosafety requirements in the laboratory. The system engineered here was able to not only detect the lentiviral particles but also to predict the viral load of the sample. This approach can be used for rapid and mass screening of infectious diseases, as it allows for analysing large number of samples simultaneously in a matter of seconds.

## Results

VNIR hyperspectral images were taken immediately after placing the samples (liquid droplets) on a supporting plate and after complete dry-up (dry residue). The diffuse optical reflectance spectra, converted to pseudo-absorbance (PA), were analysed following a pixel-based approach and integrated afterwards to obtain a per-droplet classification. They corresponded to 164 preparations (with an individual volume of 5 $\upmu$L) in two fluids: 74 samples prepared in phosphate buffered solution (PBS) and 90 in artificial saliva (AS). The numbers of pixels from samples in PBS were: 74,626 from wet droplets and 80,212 from dry residues. The numbers of pixels from samples in AS were: 92,657 from wet droplets and 94,425 from dry residues. For each fluid, the classification methods used here were trained and evaluated using the same fixed sample sets.

### Pseudo-absorbance spectra

**Figure 2:**
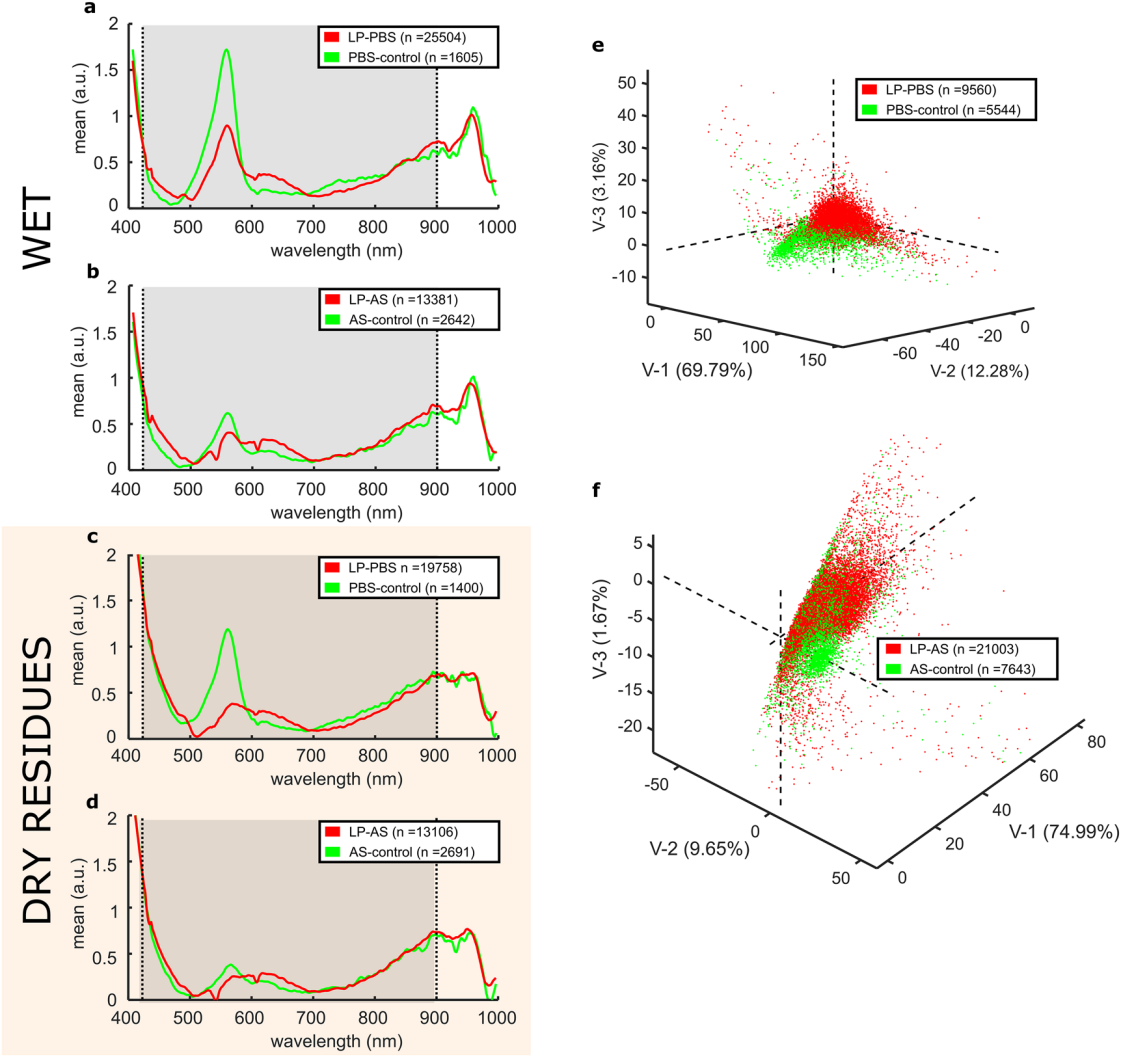
Pseudo-absorbance spectra. (**a,b**) mean pseudo-absorbance spectra obtained from lentiviral particles resuspended in PBS (LP-PBS) or artificial saliva (LP-AS), and their respective negative controls (PBS-control and AS-control). (**c,d)** overall pseudo-absorbance spectra upon complete evaporation of the aqueous content. The concentration in (**a–d)** was 4000 TU∙µL^−1^. The shadowed area highlights the wavelength range in which shape features were more informative for the FFNN analysis. (**e)** PLS-DA three-dimensional score plot of LP-PBS *vs*. PBS-control (three latent variables and captured variances: V1 = 0.70, V2 = 0.12, V3 = 0.03). (**f**) PLS-DA three-dimensional score plot of AS-PBS vs. AS-control (three latent variables and captured variances: V1 = 0.75, V2 = 0.10, V3 = 0.02).

The first step in understanding whether or not the spectra in the VNIR range could discriminate samples with viral particles from their negative controls was to obtain the overall PA spectra of the pixels from 5-$\upmu$L droplets. This volume was chosen sufficiently small to resemble relatively large respiratory droplets, and big enough to prevent rapid evaporation. The average number of pixels per droplet was approximately 1800 pixels. Pixels corresponding to image irregularities (e.g. reflection bright spots) were manually segmented, and the overall number of pixels per droplet reduced to roughly 1200, what represents two thirds of the total imaged surface of each sample. When placed on the supporting plate, the diameter of the droplet was approximately 2 mm; thus, assuming a 2-mm-in-diameter disc model, each pixel would represent a volume of 4 nL approximately, the volume of an infectious aerosol as reported for SARS-CoV-2^16^. However, the information embedded within the spectrum of a given pixel does not only arise from that particular volume but also from neighbouring sites, as the contribution of sub-surface scattering plays a predominant role in providing differential information. We appreciated relevant differences in the spectral signatures between samples with lentiviral particles and their negative controls, both in PBS and AS preparations, as shown in Fig. 2a,b. Although less prominent, there were also differences in the spectra of their corresponding dry residues (Fig. 2c,d). This suggests that the spectral signatures carry relevant information about the viral content of the samples. However, the overlapping spectral signatures of the media and the targeted viral particles possess a high variability that requires multivariate analysis. Thus, to reduce the complexity of the information contained within the PA spectra, a PLS-DA model was built as described in the Methodology. This allowed to enhance the spectral differences as illustrated in the examples shown in Fig. 2e,f.

### Classification using PLS-DA

**Figure 3:**
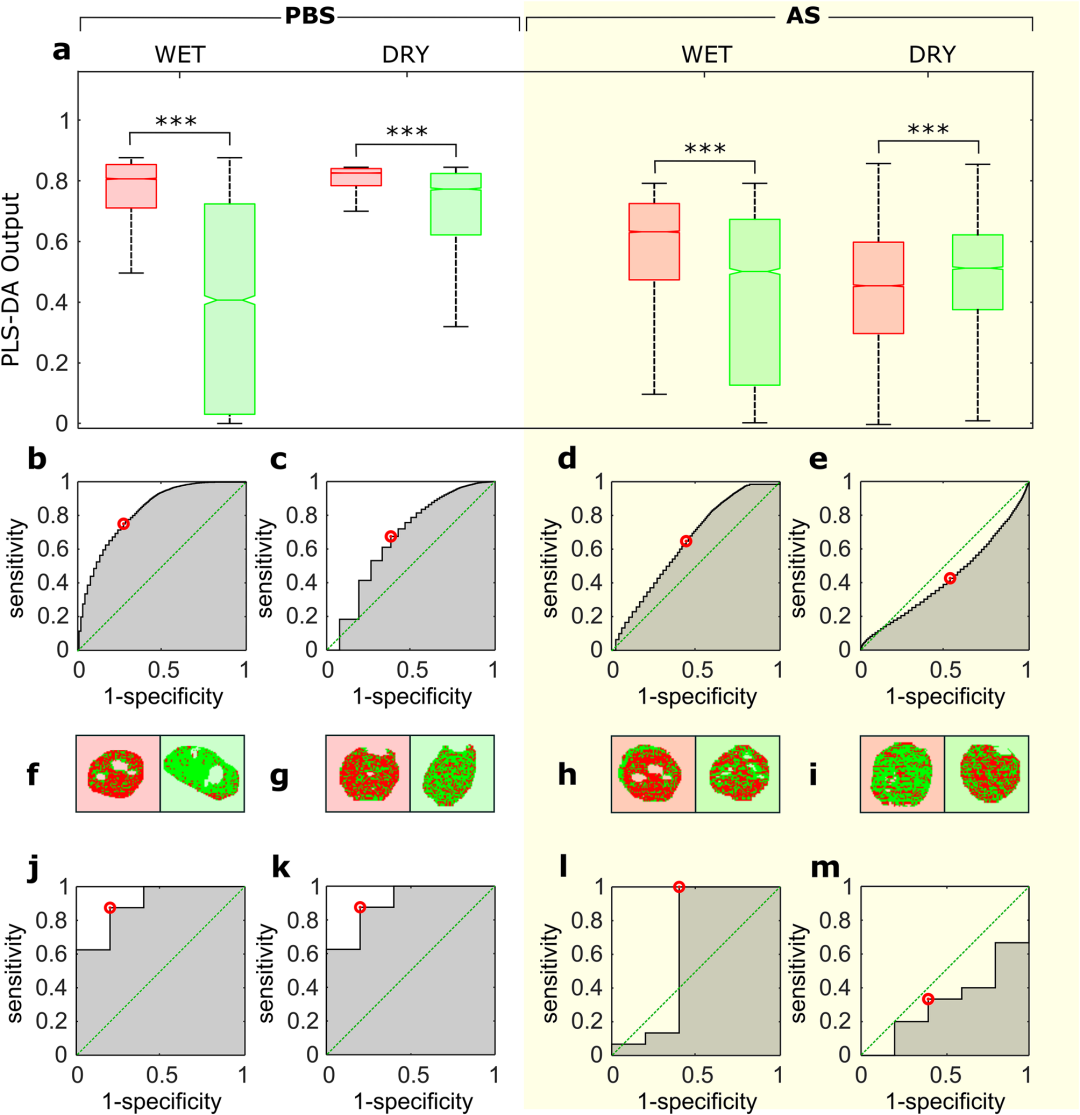
Classification of VSV-G pseudotyped lentiviral particles using PLS-DA. (**a**) median value of the PLS-DA output used for pixel classification for wet and dry samples prepared in phosphate buffer solution (PBS) and artificial saliva (AS). The boxes represent the interquartile range, and the whiskers show 2.7 times the standard deviation. Lentiviral particles (LP), denoted here in red, were prepared in PBS (LP-PBS) or in artificial saliva (LP-AS). Negative controls, represented here in green, were prepared from non-transfected culture media in PBS (PBS-control) or in artificial saliva (AS-control). ***p-value < 0.0001 from two-tailed Wilcoxon rank-sum test. (**b**) ROC curve obtained from the pixel classification of LP-PBS. (**c**) ROC curve from the pixel classification of LP-PBS obtained after complete evaporation of the water content. (**d**) ROC curve obtained from the pixel classification of LP-AS. (**e**) ROC curve from the pixel classification of LP-AS obtained after complete evaporation of the water content. (**f–i)** example of a pixel classification in a droplet. Red and green pixels denote pixels classified as positive and negative respectively. Note red and green backgrounds indicate positive droplet and its negative control respectively. (**f**) LP-PBS. (**g**) LP-PBS dry residue. (**h**) LP-AS. (**i**) LP-AS dry residue. (**j**) ROC curve obtained from droplet classification of LP-PBS. (**k**) ROC curve from droplet classification of LP-PBS obtained after complete evaporation of the water content. (**l**) ROC curve obtained from droplet classification of LP-AS. (**m**) ROC curve from droplet classification of LP-AS obtained after complete evaporation of the water content.

PLS-DA models included between 16 and 20 latent variables and retained from 95.20% up to 96.27% of data variance. The output of the PLS-DA was used for pixel classification. The Wilcoxon rank-sum test performed on these outputs revealed significant statistical differences between samples containing lentiviral particles and their non-transfected equivalent preparations (*p-value* = 0), as shown in Fig. 3a. The interquartile range overlap was more pronounced in dry residues. Nevertheless, the classifier performed better than a random classifier in all cases at pixel level, as illustrated in the receiver operating characteristic (ROC) curves shown in Fig. 3b-e. Note that a ROC curve below the diagonal line, as in Fig. 3e, represents a classifier model with an inverted output. An example of the resulting pixel classification in each case is illustrated in Fig. 3f-i. Next, to determine whether or not a droplet was taken from a sample containing viral particles, a new threshold was determined as the percentage of positive pixels within a given droplet. The ROC curves in Fig. 3j-m show an important improvement in the performance of the classifier when operating at droplet level (i.e., considering all individual pixels that constitute a given droplet), as information was gathered based on multiple observations.

The area under the ROC (AUROC), a performance metric typically used to evaluate a classifier, showed excellent accuracy in PBS preparations, both in wet droplets and dry residue (AUROC = 0.90). The accuracy of the prediction dropped importantly for samples prepared in AS. Further information on the classification performance can be found in Table 1.

**Table 1:** Classification performance of the PLS-DA method. Here, n is the sample size, Th is the value of the classification threshold, SE denotes the sensitivity, SP is the specificity, and AUROC is the area under the receiver operating characteristic curve.

		PLS-DA									
		WET					DRY RESIDUE				
		n	Th	SE (%)	SP (%)	AUROC	n	Th	SE (%)	SP (%)	AUROC
PBS	Pixel	15,104	0.71	75.0	72.8	0.82	20,527	0.80	67.5	61.9	0.66
	Droplet	13	0.65	87.5	80.0	0.90	13	0.58	87.5	80.0	0.90
AS	Pixel	28,646	0.55	64.7	55.7	0.64	31,006	0.50	42.6	46.9	0.43
	Droplet	20	0.24	100.0	60.0	0.64	20	0.49	33.3	60.0	0.32

### FFNN classification

**Figure 4:**
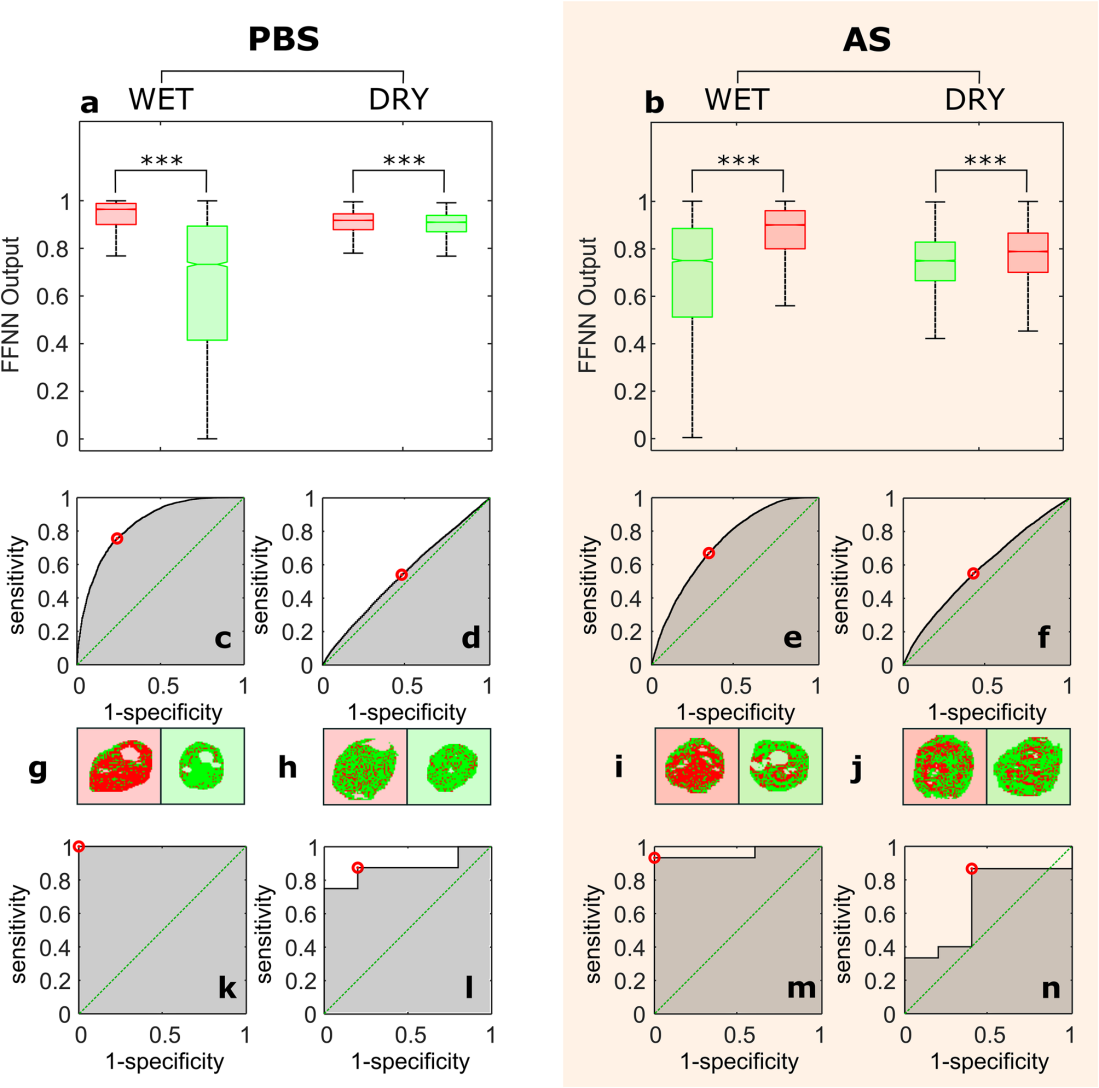
Classification of VSV-G pseudotyped lentiviral particles using FFNN. (**a**) median value of the FFNN output used for pixel classification for samples prepared in PBS. (**b**) median value of the FFNN output used for pixel classification for samples prepared in AS. (**a,b)** the boxes represent the interquartile range, and the whiskers show 2.7 times the standard deviation. Lentiviral particles (LP), denoted here in red, were prepared in PBS (LP-PBS) or in artificial saliva (LP-AS). Negative controls, represented here in green, were prepared from non-transfected culture media in PBS (PBS-control) or in artificial saliva (AS-control). ***p-value < 0.0001 from two-tailed Wilcoxon rank-sum test. (**c**) ROC curve obtained from the pixel classification of LP-PBS. (**d)** ROC curve from the pixel classification of LP-PBS obtained after complete evaporation of the water content. (**e)** ROC curve obtained from the pixel classification of LP-AS. (**f**) ROC curve from the pixel classification of LP-AS obtained after complete evaporation of the water content. (**g–j)** example of a pixel classification in a droplet. Red and green pixels denote pixels classified as positive and negative respectively. Note red and green backgrounds indicate positive droplet and its negative control respectively. (**g**) LP-PBS. (**h**) LP-PBS dry residue. (**i)** LP-AS. (**j)** LP-AS dry residue. (**k)** ROC curve obtained from droplet classification of LP-PBS. (**l)** ROC curve from droplet classification of LP-PBS obtained after complete evaporation of the water content. (**m)** ROC curve obtained from droplet classification of LP-AS. (**n)** ROC curve from droplet classification of LP-AS obtained after complete evaporation of the water content.

In order to extract the embedded information that enables detection of the viral presence, a set of 28 spectral shape descriptors was calculated in seven spectral bands of interest and fed into the FFNN, whose output took values between 0 and 1 in the same fashion as in the PLS-DA. Similarly, the Wilcoxon rank-sum test showed strong differences among the outputs of the FFNN classification obtained from preparations containing lentiviral particles and the negative controls used here, as illustrated in Fig. 4a. These differences were also observed in their dry residues (Fig. 4b). ROC curves were constructed to determine the classification thresholds, as shown in Fig. 4c-f. An example of the resulting classification of the pixels within the same droplet is shown in Fig. 4g-j.

As in the PLS-DA, a per-droplet classification was obtained by requiring a certain percentage of individual positive pixels to consider the sample as positive. Again, the droplet classification threshold was determined following the criteria previously described, as illustrated in Fig. 4k-n. As with the PLS-DA models, this integration of information from individual pixels to droplet level improved drastically the performance of the FFNN classifiers, even to 100% accuracy.

The goodness of the classification method was also assessed by computing the AUROC, which remained above 0.5 in all cases, as shown in Table 2. The sensitivity (SE) and specificity (SP) per-droplet showed similar values for preparations in PBS (SE = 100%, SP = 100%) and AS (SE = 93.3%, SP = 100%), and were substantially higher than those obtained from the PLS-DA. Regarding the analysis of the dry residues, the accuracy of the FFNN algorithm showed similar performance for PBS and AS, above a random classifier at pixel level, and substantially higher at droplet level (AUROC = 0.88 for PBS and AUROC = 0.67 for AS).

**Table 2:** Classification performance of the FFNN algorithm. Here, n is the sample size of the test set, Th is the value of the classification threshold, SE denotes the sensitivity, SP is the specificity, and AUROC is the area under the receiver operating characteristic curve.

		FFNN									
		WET					DRY Residue				
		n	Th	SE (%)	SP (%)	AUROC	n	Th	SE (%)	SP (%)	AUROC
PBS	Pixel	15,104	0.90	75.6	76.0	0.84	20,527	0.91	54.0	52.7	0.54
	Droplet	13	0.52	100.0	100.0	1.00	13	0.16	87.5	80.0	0.88
AS	Pixel	28,646	0.84	66.8	65.8	0.73	31,006	0.77	54.9	58.1	0.59
	Droplet	20	0.64	93.3	100.0	0.96	20	0.23	86.7	60.0	0.67

### Prediction of the viral load

**Figure 5:**
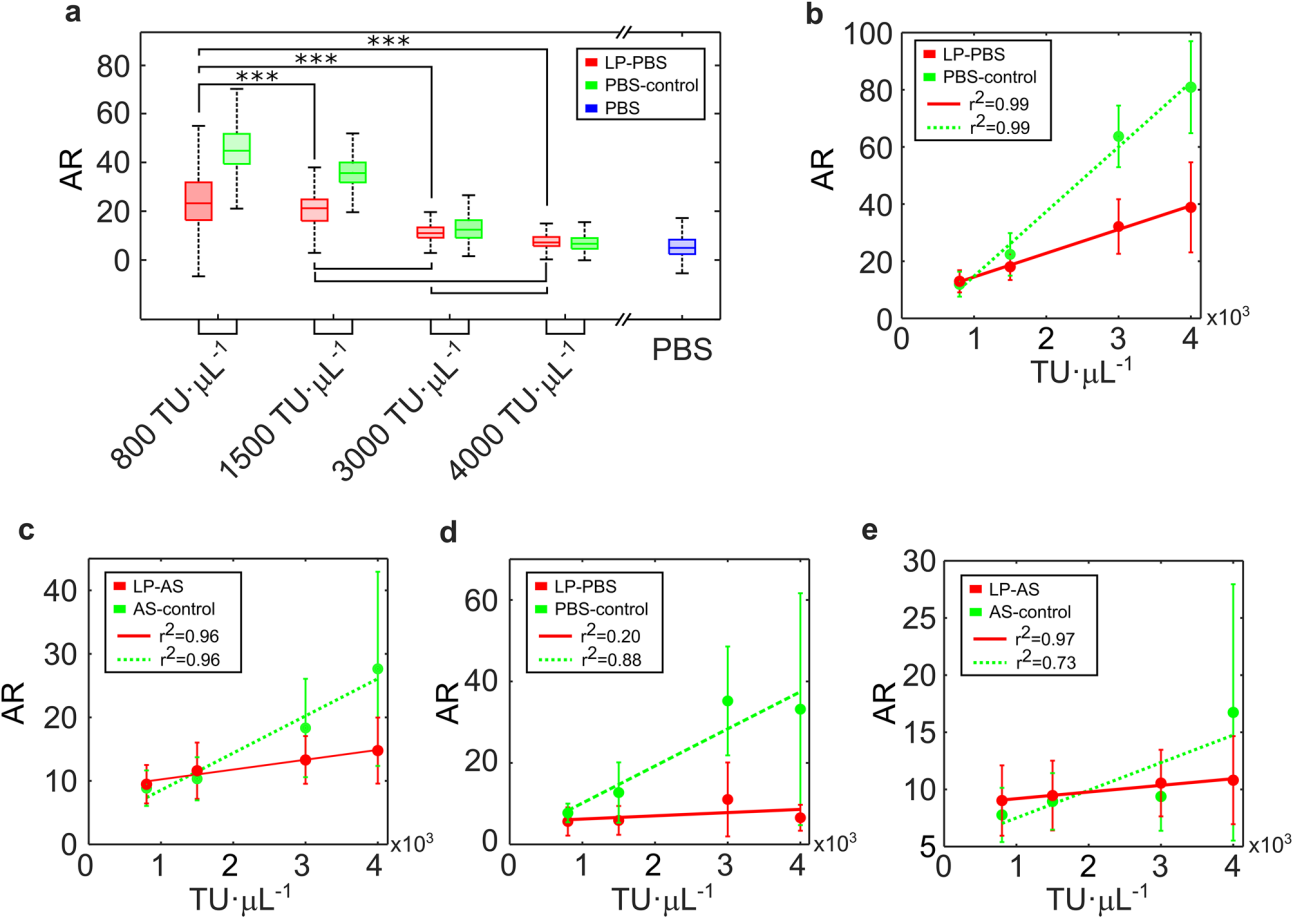
Viral load quantification. (**a)** median value of the shape feature named area ratio (AR see supplementary material), against viral load of the preparation. The boxes represent the interquartile range, and the whiskers show 2.7 times the standard deviation. Lentiviral particles in PBS (LP-PBS) and their corresponding negative controls (PBS-control). Additionally, the estimator was obtained for PBS alone. ***p-value < 0.0001 using Wilcoxon rank-sum test. (**b)** mean value of the AR obtained from LP-PBS and PBS-control. (**c)** mean value of the AR obtained from LP-AS and AS-control. (**d)** mean value of the AR obtained from LP-PBS and PBS-control upon complete evaporation of the water content. (**e)** mean value of the AR obtained from LP-AS and AS-control upon complete evaporation of the water content. (**b–e)** the error bars denote the standard deviation. The green dashed line and the red solid line represent the linear correlation, and r denotes the linear correlation coefficient.

We observed that some spectral feature descriptors could shed light about the viral load of the sample under study. In particular, the value of the area ratio (AR) descriptor (defined as the ratio between the area under the spectral curve of the sample and the area under the curve of the spectrum of the supporting plate, see supplementary material) showed statistically significant differences (Wilcoxon rank-sum test, *p-value* = 0) when evaluated at different concentrations, as illustrated in Fig. 5a. In all cases, significant differences (p-value = 0) were obtained between samples containing lentiviral particles and their negative controls prepared in equal concentrations. Furthermore, a very strong linear correlation was found between the value of the said descriptor and the viral load in fresh preparations in PBS (r^2^ = 0.99) and in AS (r^2^ = 0.96). A high correlation was also found in dry residues of AS preparations (r^2^ = 0.88), as illustrated in Fig. 5b-c,e. This linear relationship could not be established for dry residues from PBS preparations (r^2^ = 0.20), as shown in Fig. 5d. These results suggest that it is possible to quantify the viral load of fresh samples by computing key morphological descriptors in certain spectral fringes. Note here that the lowest concentration included in this analysis was 800 TU·µL^−1^. Assuming that approximately 0.1% of the virus particles that are present in the preparations are infectious^17^, this minimum viral load, expressed in -physical units- (copies·mL^−1^), would be equivalent to 8·10^8^ copies·mL^−1^. Within the wide range of viral loads of SARS-CoV-2 patients, this value corresponds to the small percentage of the so-called ‘supercarrier’ individuals, potential ‘superspreaders’ of the disease^18^.

## Discussion

Hyperspectral imaging techniques have been extensively used to rapidly identify pathogens forming biofilms on food products^19^ or to prevent the spread of plant diseases^20^ to name a few. Here we have demonstrated it is also possible to detect the presence of a virus, in this case a lab-engineered lentiviral particle, in a fluid using hyperspectral image processing techniques in the VNIR range. Although there have been successful attempts to identify viral infections in mosquitoes^11^ or plant seeds^10^ using similar methods, the previous results are limited to the analysis of ongoing infections in tissues, where histological changes or the effects of the host immune response may represent the prevalent source of information to discriminate the presence of the virus. Nonetheless, we have been able to successfully detect viral particles alone in two different media, achieving a sensitivity and a specificity in liquid droplets comparable to those reported from molecular techniques (SE = 100%, SP = 100% in PBS, SE = 93.3%, SP = 100% in AS). At present, the gold standard for the detection of a virus is the polymerase chain reactions (PCR) test. However, with the advent of the global pandemic caused by the SARS-CoV-2 virus, a number of new molecular-based diagnostic technologies have emerged^21,22^, allowing for a substantial reduction of turnaround times. A significant advantage of VNIR hyperspectral imaging detection over traditional diagnostic techniques relies on the possibility of performing non-contact, reagent-free analysis of a large number of samples simultaneously. We prepared the system to image between 9 and 25 droplets over an area of 9 × 8 cm^2^, but this technology can be easily scaled to increase the field of view, thus allowing for the analysis of numerous samples concurrently. Note that using a standard personal computer, the generation of numerical models can take several hours. However, once these models are built, the average processing time per droplet is approximately one minute for PLS-DA, and can be substantially reduced when using the FFNN algorithms described here.

It might also have the potential to be adapted for use in personal devices (e.g. *smartphones*) with adequate optical adapters^23^. Furthermore, when compared to other optical spectroscopic techniques that rely on the use of a single beam of light, hyperspectral imaging provides information redundancy by accounting for a collection of pixels within a given sample. Note that even when the performance of the classifier at pixel level was slightly above a random classifier, there was a substantial improvement at droplet level. This suggests that if the spectral signature of a given virus contains distinctive information, this can be extracted and enhanced by analysing multiple samples.

The envelope of the lentiviral particles used in this study was VSV-G glycoprotein. This glycoprotein confers some optical properties that may be responsible for its spectral signature. Nevertheless, this study cannot discern whether the information is arising at molecular level or from their aggregations or structural properties, and the corresponding virological and biochemical studies remain open. It is also important to note that these viral particles have a diameter between 80 nm and 120 nm^24,25^, which is approximately three times smaller than the shortest wavelength within the visible range (i.e. blue light). Therefore, if the information is embedded in the scattered light, the PA spectra obtained from other viral species might also possess distinct features that would enable their optical identification. If that is confirmed, there are numerous applications for this technology. However, the challenge lies with finding spectral features that are substantially different from the background and the environment, that is, the supporting plate and the fluid media.

In this study, samples were classified using two different independent methods. The performance of both techniques were similar, a point that supports the hypothesis underpinning this work. In addition, the statistically significant differences found in the value of one of the spectral feature descriptors across different sample concentrations revealed that there was enough information for quantifying the viral load. It is therefore likely that a combination of different spectral descriptors, when used in an ANN, could also be exploited to further refine the quantification results reported here, as the combination of multiple sources of information can reveal these hidden details.

We also sought to determine whether this imaging method could be used for the screening of viral contamination on fomites. Although the performance of the classifier was in general lower in dry residues compared to liquid samples, the technique showed values of the AUROC between 0.67 and 0.88. In particular, in AS, a medium that mimics human fluids, the sensitivity thus obtained was 87.5% (SP = 60.0%). Note that this study presents a relatively unbalanced number of positive and negative samples leaving room for further improvements. A PLS-DA model built with unbalanced datasets substantially improves with balanced data^26^.The results reported here support the use of this technology, not only for the screening of fluid samples obtained from a large number of subjects but also for a potential pre-screening of inanimate objects which may be subjected to viral contamination.

## Materials and methods

### VSV-G pseudotyped lentiviral particles

For the production of the lentiviral particles, 293 T cells were transfected with a lentiviral plasmid coding for ZsGreen, a plasmid coding for viral envelope VSGV-g protein (under the control of the human cytomegalovirus promoter) and a plasmid encoding the Tat, Gag-Pol and Rev genes required to construct a 2nd-generation lentiviral system^27,28^. HEK cells were cultured in Gibco Dulbecco's Modified Eagle Medium (DMEM) supplemented with 10% foetal bovine serum (Biowest, Nuaillé, France) and a combination of penicillin (100 UI·mL^−1^) and streptomycin (100 μg·mL^−1^) (MilliporeSigma, Missouri, USA). After transfection, cells were incubated at 37ºC, 5% CO2 for 48 h. Next, the culture medium containing viral particles was collected and Lenti-X reagent (Takara Bio Inc, Shiga, Japan) was added to concentrate viral particles through precipitation. The mixture was then incubated at 4ºC followed by centrifugation at 1500 G for 45 min at 4ºC.The supernatant was then removed, and the precipitate stored at -80ºC for later use. As a negative control, un-transfected HEK 293 T cells were cultured and the medium was collected, precipitated with LentiX, and aliquoted as described above.

One hour prior to recording the reflectance spectra, a viral aliquot was thawed and resuspended in either phosphate buffered saline (MilliporeSigma, Missouri, USA) or 1700–0305 artificial saliva (Pickering Laboratories, California, USA). From an initial stock titer of 20·10^3^ transducing units (TU)·$\upmu$L^−1^, serial dilutions were prepared in the corresponding media to obtain the following set of concentrations: [500, 800, 1000, 1500, 2000, 2500, 3000, 3500, 4000] TU·$\upmu$L^−1^.

### Negative controls

Droplets of the same fluid (PBS or AS) with the same concentrations of virus culture medium (DMEM) and Lenti-X reagent but without lentiviral particles were used as negative controls. In addition, droplets of pure fluids were included for comparison.

### Cell cultures

Human Embryonic Kidney (HEK) Lenti-XTM 293 T cell lines (Clontech) were maintained in Dulbecco’s modified Eagle’s medium (DMEM) supplemented with 10% (v/v) heat inactivated Fetal Bovine Serum (FBS), 2 mM L-glutamine, 10 mM Hepes, 1% (v/v) sodium pyruvate, 50 μM 2-mercaptoethanol, 100 U·mL^-1^ penicillin and 100 μg·mL^-1^ streptomycin at 37°C, 10% CO2.

### Hyperspectral imaging setup and calibration

Spectral information was obtained between 406.62 nm and 996.46 nm using an A-Series VNIR hyperspectral imaging system (Headwall, Massachusetts, USA), in 810 bands with 0.74 nm of nominal spectral resolution. These wavelengths range from the lower limit of the visible (VIS) to the near infra-red (NIR) band of the electromagnetic spectrum. The camera was mounted 30 cm over the sample plane on an A-LST0750-C (Zaber, Vancouver, Canada) motorised linear stage to allow scanning of the samples, and a 35-mm LM35HC lens (KOWA, Saitama, Japan) was used to provide optimal focusing. An area of 9 × 8 cm^2^ was scanned to ensure light intensity remained approximately homogenous over the sample. The image had a resolution of 1275 pixels by 1000 pixels; therefore, the area of a single pixel was approximately of 70 × 80 $\upmu$m^2^. Optical reflectance signals at each spectral wavelength were codified using 8 bits.

The samples were illuminated using two ASD Illuminator halogen light sources (Malvern Pabalytical, Worcestershire, UK) mounted symmetrically 35 cm above the sample plane with their emission axes forming a 60-degree angle with the sample plane. The colour temperature of the light source was 3100 K and the emission spectrum ranged between 350 nm and 2500 nm. The experiments were carried out under low ambient illumination to mimic a potential point-of-care testing environment. Illumination irradiance was measured in a square grid pattern of 16 points covering the area of the sample supporting plate using a radiometer (HD 2102.2 with a LP 471 Probe, Delta OHM srl, Padua, Italy). The contribution from ambient light was 1.03 ± 0.05 W∙m^−2^. When the experimental illumination sources were connected, the average irradiance on the supporting plate was 252 ± 28 W∙m^−2^.

The system was then linearly calibrated between a white and a dark reference. The white reference was obtained from a 3.62-inch Spectralon white reference (Labsphere, New Hampshire, USA). The dark reference was generated by blocking the lens using the lens cap provided by the manufacturer. Note the dark reference contains the background noise generated by the photodetector.

### Preparation of samples and imaging

The samples were imaged at room temperature under ambient conditions (relative humidity varying between 40% and 60%). Using a micropipette, 5-$\upmu$L droplets were deposited onto the surface of an approximately 22 mm × 22 mm polytetrafluoroethylene sheet (BSH, Seville, Spain) with a thickness of 1 mm (supporting plate). Each preparation contained between 9 and 25 droplets distributed in a square mosaic pattern to facilitate off-line digital segmentation. The samples were immediately positioned within the field of view of the imaging system over a 10-mm-thick wood sheet. The reflectance spectra were then recorded. Following complete evaporation of the aqueous content, a second reflectance spectra dataset was obtained from the dry residues.

### Computer equipment

Processing units were standard, high-end personal computers (128 Gb RAM, Intel® Core(TM) i9-10980XE CPU 3.00 GHz) running under Windows® 10 Pro, 64 bits.

### Sample sets

A total of 164 preparations (74 samples in PBS and 90 in AS) were analysed. The sample distribution among the different experimental groups is shown in Table 3. These same test groups were used to evaluate both the PLS-DA and FFNN classifiers. See supplementary material for a detailed description of positive and negative samples for each fluid and concentration.

**Table 3:** Number of droplets analysed using the PLS-DA and FFNN methods.

Method	Experimental groups	Fluid samples					
		PBS			AS		
		Positive	Control	Total	Positive	Control	Total
*PLS-DA*	Training	52	9	61	58	12	70
	Test	8	5	13	15	5	20
	Total	60	14	74	73	17	90
*FFNN*	Training	40	5	45	46	8	54
	Validation	12	4	16	12	4	16
	Test	8	5	13	15	5	20
	Total	60	14	74	73	17	90

### Image pre-processing

Original spectra of hyperspectral images were converted into pseudo-absorbance (PA) by computing the logarithm of the inverse of the reflectance (R) spectra (PA = log (1/R)). A unit offset was added to avoid zero-singularities in the logarithmic transform. A standard white-dark calibration was then applied. Using the hyperspectral imaging software Evince (Prediktera, Umeå, Sweden), a red–green–blue (RGB) subset of the hyperspectral cube was used to segment the content of each droplet. A conservative approach was adopted to discard peripheral pixels. Next, a principal component analysis (PCA) with two components was performed on the spectra of all pixels within a droplet to discard outlier pixels, typically flare-affected pixels. The spectra were then normalised using the standard normal variate (SNV) transform. A Savitzky-Golay filter was applied afterwards for smoothing, and a correction of the baseline was performed.

### Partial Least-Squares Discriminant Analysis (PLS-DA)

A PLS-DA model^29^ was constructed using the individual spectra of all pixels of training samples as input, and retaining a number of latent variables sufficient to capture over 95% of data variance using PLS Toolbox 8.6 (Eigenvector Research Inc, Washington, USA) running under Matlab R2020b (The Mathworks Inc., Massachusetts, USA). It generated an output variable with values between 0 and 1 for the classification of each pixel in the test set. Additionally, we re-analysed the data using the mean PA spectra for each droplet (see supplementary material for details).

### Feed-Forward Neural Network (FFNN)

We constructed an ANN following a pixel-based approach^30^. The input of the network consisted of the values of 28 spectral feature descriptors obtained in the seven spectral bands of interest (between 415 nm and 900 nm). These descriptors quantify representative morphological features (amplitude, length, curve ringing, curvature, compression, kurtosis, horizontality, area and comparisons with the background reference) of every individual spectrum (see the supplementary material for a full description). The output of the network, a value comprised between 0 and 1, was then used for pixel classification. The spectra were classified using an FFNN implemented in Matlab R2020b (The Mathworks Inc., Massachusetts, USA) with sigmoid hidden and softmax output neurons. The FFNN consisted of 20 hidden and one output layers. The number of neurons (196) in each hidden layer was set as the product of the number of spectral descriptors and bands of interest (28 × 7 = 196). Other configurations were discarded after empirical evaluation for best outcomes. To find the optimal weights, the supervised training was carried out using the scaled conjugate gradient backpropagation, limiting the number of iterations to 1000 to avoid overfitting. The performance of the network was tested by evaluating the mean square error. Training, validation and test sets were constructed using random splits of independent samples.

### Data analysis

The non-parametric two-tailed Wilcoxon rank-sum test was performed on the outputs of the PLS-DA and the FFNN models, and on the spectral feature descriptor used for quantification of the viral load. To determine classification thresholds, ROC curves were analysed. The cut-off value was chosen to maximise both, sensitivity and specificity. This value was determined as the point of the curve with minimum quadratic distance to the upper left corner of highest sensitivity and specificity^31^. To assess the goodness of the classifier, the AUROC was also computed, and the corresponding confusion matrix was calculated. From values of true positive (TP), true negative (TN), false positive (FP) and false negative (FN) classifications, sensitivity (SE) and specificity (SP) were then obtained as follows: SE = TP/(TP + FN), SP = TN/(TN + FP). Statistical significance was considered at the 95% confidence level. P-values below 0.0001 were considered as zero.

## Supplementary Information

Supplementary Information.

## Data Availability

All data needed to evaluate the findings are present in the paper and the supplementary materials. Additional data related to this work are available from the authors under reasonable request.
