# Supplementary material for: Hyperspectral image processing for the identification and quantification of lentiviral particles in fluid samples
Source: Sci Rep. 2021 Aug 10;11:16201. doi: 10.1038/s41598-021-95756-3 (PMC8355230; doi:10.1038/s41598-021-95756-3)
Supplement: Supplementary file 1 — Supplementary Information. [file 41598_2021_95756_MOESM1_ESM.docx]

**SUPPLEMENTARY INFORMATION**

**Article: “Hyperspectral image processing for the identification and quantification of lentiviral particles in fluid samples”.**

**Authors:**

Emilio Gomez-Gonzalez^1,2^, Beatriz Fernandez-Muñoz^3^, Alejandro Barriga-Rivera^1,4^, Jose Manuel Navas-Garcia^5^, Isabel Fernandez-Lizaranzu^1,2^, Francisco Javier Munoz-Gonzalez^1^, Ruben Parrilla-Giraldez^6^, Desiree Requena-Lancharro^1^, Manuel Guerrero-Claro^1^, Pedro Gil-Gamboa^1^, Cristina Rosell-Valle^2,3^, Carmen Gomez-Gonzalez^7^, Maria Jose Mayorga-Buiza^2,8^, Maria Martin-Lopez^2,3^, Olga Muñoz^9^, Juan Carlos Gomez Martin^9^, Maria Isabel Relimpio Lopez^10,11^, Jesus Aceituno-Castro^9,12^, Manuel A. Perales-Esteve^13^, Antonio Puppo-Moreno^7^, Francisco Jose Garcia Cozar^14^, Lucia Olvera-Collantes^15^, Silvia de los Santos-Trigo^16^, Emilia Gomez^17^, Rosario Sanchez Pernaute^3^, Javier Padillo-Ruiz^2,18^, Javier Marquez-Rivas^2,19,20^.

1. Department of Applied Physics III, School of Engineering, Universidad de Sevilla, 41092 Sevilla, Spain
2. Institute of Biomedicine of Seville, 41013 Sevilla, Spain
3. Unidad de Producción y Reprogramación Celular (UPRC), Red Andaluza de Diseño y Traslación de Terapias Avanzadas, 41092 Sevilla. Spain
4. School of Biomedical Engineering, The University of Sydney, NSW 2006, Australia.
5. EOD-CBRN Group, Spanish National Police, 41011 Sevilla, Spain.
6. Technology and Innovation Centre, Universidad de Sevilla, 41012 Sevilla, Spain.
7. Service of Intensive Care, University Hospital ‘Virgen del Rocio’, 41013 Sevilla, Spain.
8. Service of Anaesthesiology, University Hospital ‘Virgen del Rocio’, 41013 Sevilla, Spain.
9. Instituto de Astrofísica de Andalucía, CSIC, 18008 Granada, Spain.
10. Department of Ophthalmology, University Hospital ‘Virgen Macarena’, 41009 Sevilla, Spain.
11. OftaRed, Institute of Health ‘Carlos III’, 28029 Madrid, Spain.
12. Centro Astronomico Hispano Alemán, 04550 Almeria, Spain.
13. Department of Electronic Engineering, School of Engineering, Universidad de Sevilla, 41092 Sevilla, Spain.
14. Department of Biomedicine, Biotechnology and Public Health, University of Cadiz, 11003 Cadiz, Spain.
15. Instituto de Investigación e Innovación Biomedica de Cádiz (INIBICA), 11009 Cadiz, Spain.
16. Corporación Tecnológica de Andalucía, 41092 Sevilla, Spain.
17. Joint Research Centre, European Commission, 41092 Sevilla, Spain.
18. Department of General Surgery, University Hospital ‘Virgen del Rocío’, 41013 Sevilla, Spain.
19. Service of Neurosurgery, University Hospital ‘Virgen del Rocío’, 41013 Sevilla, Spain.
20. Centre for Advanced Neurology, 41013 Sevilla, Spain.

**Correspondence:**

Prof. Emilio Gomez-Gonzalez

Department of Applied Physics III

School of Engineering, Universidad de Sevilla

Camino de los Descubrimientos s/n, 41092 Sevilla, Spain,

[egomez@us.es](mailto:egomez@us.es)

***Spectral feature descriptors***

The feature descriptors here provide a quantitative assessment of the morphological characteristics of the pseudo-absorbance (PA) spectra (see Fig. 6). These estimators together provide the insights of the spectral signatures analyzed by the data-driven approaches implemented in this study. For each spectrum, all descriptors are to be applied within certain spectral sub-bands, denoted here by their initial-end wavelength range${[\lambda}_{i},\lambda_{j}]$. Tables Is and IIs show, respectively, the spectral characteristics and the bands of interest where the feature descriptors were calculated:

| SPECTRAL CHARACTERISTICS | Descriptors |
| --- | --- |
| Amplitude | F1 – F5 |
| Length | F6 – F8 |
| Curve ringing | F9 – F10 |
| Curvature | F11 – F13 |
| Compression | F14 – F18 |
| Kurtosis | F19 – F20 |
| Horizontality | F21 – F24 |
| Triangle area | F25 |
| Angle with respect to a reference | F26 |
| Comparison with respect to a reference | F27 – F28 |
| Area ratio with respect to a reference | AR |

Table Is. Geometrical spectral characteristics covered by descriptors. The PA spectrum of the sample supporting plate includes the potential contributions from the underlying substrate (a 10 mm-thick wood sheet). It is defined as “background” and used as reference for the calculation of descriptors.

| BAND | SPECTRAL RANGE (nm) |
| --- | --- |
| R1 | 415 - 450 |
| R2 | 425 - 440 |
| R3 | 480 - 510 |
| R4 | 510 - 600 |
| R5 | 525 - 560 |
| R6 | 600 - 616 |
| R7 | 700 - 900 |

Table IIs. Spectral bands of interest (for descriptors F1-F28).

***Spectral feature descriptors for FFNN.***

All 28 (F1 – F28) descriptors proposed here were computed on the seven spectral ranges shown in Table IIs. A total of 28 x 7 = 196 values were thus generated and served as the input of the FFNN used for the classification of the PA spectra at pixel level.

- **F1-F5:** these descriptors provide a comparison between the **amplitude** of the spectral curve and that of the line obtained by linearly interpolating the spectrum between the two ends of the spectral range under analysis (see Fig. 6 a,b).
  - F1 – ratio between the value of the local minimum of the spectrum and that of linear interpolation at the same wavelength.
  - F2 – difference between the value of the local minimum of the spectrum and that of the linear interpolation at the same ordinate.
  - F3 – absolute value of F2.
  - F4 – ratio (percentage) between the value of F3 and the difference between the spectral minimum and maximum.
  - F5 – qualitative description of the amplitude from F4 as very low (1), low (2), normal (3) high (4) and very high (5).
- **F6-F8:** these descriptors provide an estimation of the **length** of the spectral curve (see Fig. 6b).
  - F6 – linear distance between the two end points of the spectral curve.
  - F7 – length of the spectral curve.
  - F8 – length of the 10th–order interpolation polynomial.
- **F9-F10:** these spectral features assess the **curve ringing** (see Fig. 6b).
  - F9 – ratio (percentage) between the length of the curve (F7) and the length of the 10th–order interpolation polynomial (F8).
  - F10 – qualitative description of the ringing: very low (1), low (2), normal (3) high (4) and very high (5).
- **F11-F13:** provide an assessment of the **curvature** of the spectral fringe of interest by comparing the length of the spectral curve to that of the linear interpolation between the two ends of the spectral interval (see Fig. 6b). For that purpose, here we determined whether the length of the curve was better approximated by a linear interpolation or by a 10th-order polynomial interpolation. Then, a conditional statement was applied to determine whether the curve was compared to the polynomial or the linear interpolation.
  - F11 – Boolean variable that determines if there exists a local minimum in the interval.
  - F12 – Boolean variable that determines whether polynomial or linear interpolation is to be used for comparison in the next descriptor.
  - F13 – ratio between the length of the spectral curve (F7) and that of the interpolation (F6 or F8). The decision is made based on the value of F12.
- **F14-F18:** these spectral features provide an estimation of the **compression** of the spectrum, defined as the difference between the maximum and minimum values of the pseudo-absorbance within the spectral range of interest (see Fig. 6b).
  - F14 – qualitative description of the curvature: concave (1) or convex (2).
  - F15 – qualitative description of the compression of the curve in the x axis: low (1), normal (2), high (3) and very high (4).
  - F16 – qualitative description of the compression of the curve in the y axis: low (1), normal (2), high (3) and very high (4).
  - F17 – qualitative overall compression of the spectral curve as per F16 and F17 combined: low (1), normal (2), high (3) and very high (4).
  - F18 – qualitative description of the symmetry of the curve relative to the centre of the spectral fringe: low (1), normal (2), high (3) and very high (4).
- **F19-F20:** describe the shape of the spectral band using the **kurtosis** test (assuming it derives from a stochastic process).
  - F19 – kurtosis test value.
  - F20 – qualitative description of the symmetry based on the kurtosis test: mesokurtic (1), leptokurtic (2) and platykurtic (3).
- **F21-24:** these descriptors relate to the degree of **horizontality** of the spectrum in the band under analysis (see Fig. 6c).
  - F21 – slope of the regression line.
  - F22 – absolute value of F21.
  - F23 – y-intercept value of the regression line.
  - F24 – qualitative assessment of the horizontality: high (1), normal (2), low (3), and very low (4).
- **F25:** using the geometrical Heron’s formula, this descriptor provides the **area of the triangle** described by the two ends of the spectral band under analysis and the central point of the spectral curve (see Fig. 6d).
- **F26:** **angle** (in degrees) between the regression line of the PS spectrum of the sample under study and the regression line of the overall PA spectrum of the supporting plate (background) (see Fig. 6e).
- **F27-28:** these descriptors provide an **overall comparison** of a given spectrum to the background. In other words, they determine the proportion of the wavelength range in which one curve is above the other (see Fig. 6f).
  - F27 – percentage of the wavelength range in which the PA spectrum of the sample is above the PA spectrum of the supporting plate.
  - F28 – Boolean value. Takes value 1 if F27 ≥ 50%.

***Spectral feature descriptor for viral load determination.***

An additional spectral feature was defined to quantify the viral load. The descriptor, named here area ratio (AR), is defined as the ratio between the area under the spectral curve and the area under the spectral curve of the background, that is, that of the sample supporting plate, as shown in Fig. 6. This descriptor was applied in the optical range between 547 nm and 580 nm for samples prepared in PBS, and between 480 nm and 580 nm for samples prepared in AS.

| 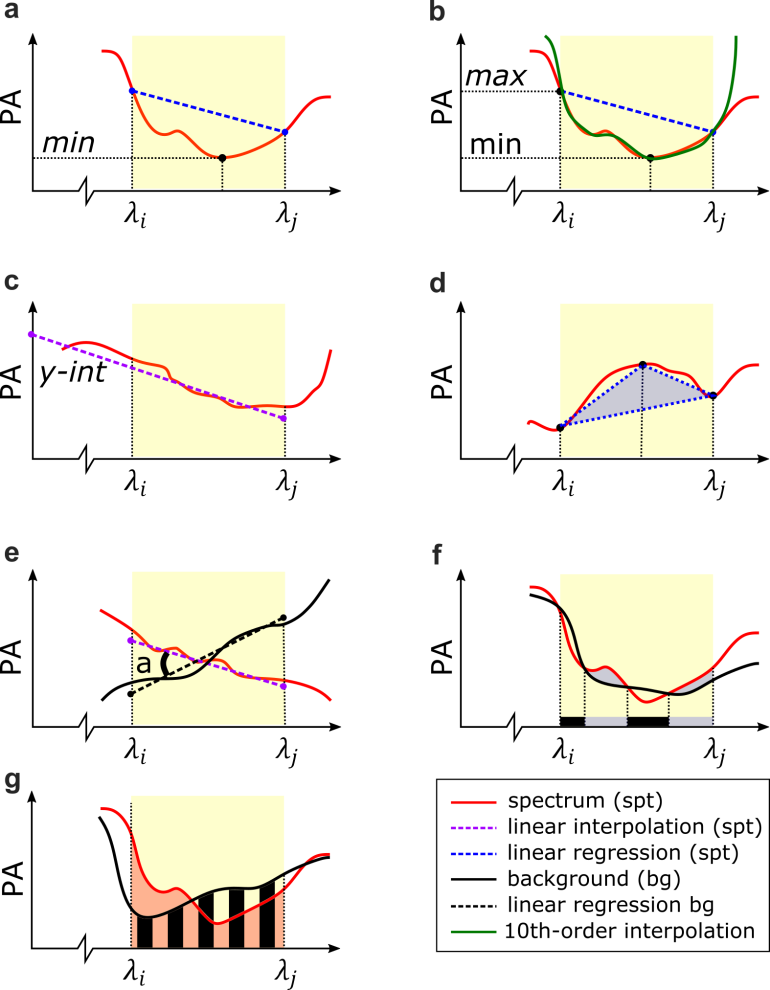 | **Fig. 6 –** **Descriptors of spectral features**.  (**a**) pseudo-absorbance (PA) spectrum (spt) of a sample with linear interpolation. (**b**) PA spectrum with linear interpolation and 10^th^-order polynomial interpolation. (**c**) PA spectrum with linear regression. (**d**) PA spectrum with inscribed triangle between the two ends of the spectral fringe of interest and the middle point of the interval. (**e**) PA spectrum of the sample and the background (bg) with linear interpolation for the calculation of the angle α. (**f**) PA spectrum of the sample and the background showing the wavelength ranges when the background curve is above the sample (black band) and *vice versa* (grey band). (**g**) area under the PA spectral curve of the sample (red) and the background (bars). (**a-g**) the wavelength range of interest is highlighted in yellow. λ_i_ and λ_j_ represent the start and end point of the spectral fringe. |
| --- | --- |

***Description of droplet and pixel distribution among experimental groups.***

Table IIls, IVs, Vs and VIs show the detailed distribution of droplets –and the corresponding number of pixels- for both processing method, fluids, concentrations and status (wet and dry) of the samples.

| **Method** | **Experimental Groups** | | **Number of positive droplets and pixels** | | | | | | | | | | |
| --- | --- | --- | --- | --- | --- | --- | --- | --- | --- | --- | --- | --- | --- |
|  |  |  | **PBS** | | | | | | | | | | |
|  |  |  | ***C0*** | ***C1*** | ***C2*** | ***C3*** | ***C4*** | ***C5*** | ***C6*** | ***C7*** | ***C8*** | ***C9*** | ***TOTAL*** |
| ***PLS-DA*** | ***Training*** | ***Droplet (T0/T10)*** | 1 | 24 | 9 | - | - | - | 9 | - | 9 | - | 52 |
|  |  | ***Pixels (T0)*** | 581 | 25504 | 10532 | - | - | - | 8536 | - | 8205 | - | 53358 |
|  |  | ***Pixels (T10)*** | 520 | 19758 | 10377 | - | - | - | 11065 | - | 10974 | - | 52694 |
|  | ***Test*** | ***Droplet (T0/T10)*** | - | - | - | 2 | 1 | 2 | - | 1 | - | 2 | 8 |
|  |  | ***Pixels (T0)*** | - | - | - | 2521 | 1193 | 2418 | - | 1334 | - | 2094 | 9560 |
|  |  | ***Pixels (T10)*** | - | - | - | 3352 | 1702 | 3185 | - | 1770 | - | 2926 | 12935 |
|  | **TOTAL** | ***Droplet (T0/T10)*** | 1 | 24 | 9 | 2 | 1 | 2 | 9 | 1 | 9 | 2 | 60 |
|  |  | ***Pixels (T0)*** | 581 | 25504 | 10532 | 2521 | 1193 | 2418 | 8536 | 1334 | 8205 | 2094 | **62918** |
|  |  | ***Pixels (T10)*** | 520 | 19758 | 10377 | 3352 | 1702 | 3185 | 11065 | 1770 | 10974 | 2926 | **65629** |
| ***FFNN*** | ***Training*** | ***Droplet (T0/T10)*** | 1 | 21 | 6 | - | - | - | 6 | - | 6 | - | 40 |
|  |  | ***Pixels (T0)*** | 581 | 22525 | 6987 | - | - | - | 6341 | - | 6211 | - | 42645 |
|  |  | ***Pixels (T10)*** | 520 | 17055 | 7372 | - | - | - | 8746 | - | 8279 | - | 41972 |
|  | ***Validation*** | ***Droplet (T0/T10)*** | - | 3 | 3 | - | - | - | 3 | - | 3 | - | 12 |
|  |  | ***Pixels (T0)*** | - | 2979 | 3545 | - | - | - | 2195 | - | 1994 | - | 10713 |
|  |  | ***Pixels (T10)*** | - | 2703 | 3005 | - | - | - | 2319 | - | 2695 | - | 10722 |
|  | ***Test*** | ***Droplet (T0/T10)*** | - | - | - | 2 | 1 | 2 | - | 1 | - | 2 | 8 |
|  |  | ***Pixels (T0)*** | - | - | - | 2521 | 1193 | 2418 | - | 1334 | - | 2094 | 9560 |
|  |  | ***Pixels (T10)*** | - | - | - | 3352 | 1702 | 3185 | - | 1770 | - | 2926 | 12935 |
|  | **TOTAL** | ***Droplet (T0/T10)*** | 1 | 24 | 9 | 2 | 1 | 2 | 9 | 1 | 9 | 2 | 60 |
|  |  | ***Pixels (T0)*** | 581 | 25504 | 10532 | 2521 | 1193 | 2418 | 8536 | 1334 | 8205 | 2094 | **62918** |
|  |  | ***Pixels (T10)*** | 520 | 19758 | 10377 | 3352 | 1702 | 3185 | 11065 | 1770 | 10974 | 2926 | **65629** |

**Table IIIs.** PBS positive droplets with each concentration in wet samples (T0) and dry residue (T10). Note that in some cases the number of segmented pixels is different between the wet sample and the dry residue. Initial stock titer C0 = 20·10^3^ TU·μL^-1^. Serial dilutions C1 = 500 TU·μL^-1^, C2 = 800 TU·μL^-1^, C3 = 1000 TU·μL^-1^, C4 = 1500 TU·μL^-1^, C5 = 2000 TU·μL^-1^, C6 = 2500 TU·μL^-1^, C7 = 3000 TU·μL^-1^, C8 = 3500 TU·μL^-1^, C9 = 4000 TU·μL^-1^.

| **Method** | **Experimental Groups** | | **Number of positive droplets and pixels** | | | | | | | | | |
| --- | --- | --- | --- | --- | --- | --- | --- | --- | --- | --- | --- | --- |
|  |  |  | **AS** | | | | | | | | | |
|  |  |  | ***C1*** | ***C2*** | ***C3*** | ***C4*** | ***C5*** | ***C6*** | ***C7*** | ***C8*** | ***C9*** | ***TOTAL*** |
| ***PLS-DA*** | ***Training*** | ***Droplet (T0/T10)*** | 15 | 14 | - | - | - | 14 | - | 15 | - | 58 |
|  |  | ***Pixel (T0)*** | 13381 | 12755 |  |  |  | 11315 |  | 16594 |  | 54045 |
|  |  | ***Pixel (T10)*** | 13106 | 12418 |  |  |  | 11724 |  | 16295 |  | 53543 |
|  | ***Test*** | ***Droplet (T0/T10)*** | - | - | 3 | 3 | 3 | - | 3 | - | 3 | 15 |
|  |  | ***Pixel (T0)*** | - | - | 4288 | 4467 | 4287 | - | 4159 | - | 3802 | 21003 |
|  |  | ***Pixel (T10)*** | - | - | 4940 | 4472 | 5270 | - | 4231 | - | 4059 | 22972 |
|  | **TOTAL** | ***Droplet (T0/T10)*** | 15 | 14 | 3 | 3 | 3 | 14 | 3 | 15 | 3 | 73 |
|  |  | ***Pixel (T0)*** | 13381 | 12755 | 4288 | 4467 | 4287 | 11315 | 4159 | 16594 | 3802 | **75048** |
|  |  | ***Pixel (T10)*** | 13106 | 12418 | 4940 | 4472 | 5270 | 11724 | 4231 | 16295 | 4059 | **76515** |
| ***FFNN*** | ***Training*** | ***Droplet (T0/T10)*** | 12 | 11 | - | - | - | 11 | - | 12 | - | 46 |
|  |  | ***Pixel (T0)*** | 10642 | 10367 | - | - | - | 8980 | - | - | - | - |
|  |  | ***Pixel (T10)*** | 10538 | 10356 | - | - | - | 9697 | - | - | - | 44840 |
|  | ***Validation*** | ***Droplet (T0/T10)*** | 3 | 3 | - | - | - | 3 | - | 3 | - | 12 |
|  |  | ***Pixel (T0)*** | 2739 | 2388 | - | - | - | 2335 | - | 2444 | - | 9906 |
|  |  | ***Pixel (T10)*** | 2568 | 2062 | - | - | - | 2027 | - | 2046 | - | 8703 |
|  | ***Test*** | ***Droplet (T0/T10)*** | - | - | 3 | 3 | 3 | - | 3 | - | 3 | 15 |
|  |  | ***Pixel (T0)*** | - | - | 4288 | 4467 | 4287 | - | 4159 | - | 3802 | 21003 |
|  |  | ***Pixel (T10)*** | - | - | 4940 | 4472 | 5270 | - | 4231 | - | 4059 | 22972 |
|  | **TOTAL** | ***Droplet (T0/T10)*** | 15 | 14 | 3 | 3 | 3 | 14 | 3 | 15 | 3 | 73 |
|  |  | ***Pixel (T0)*** | 13381 | 12755 | 4288 | 4467 | 4287 | 11315 | 4159 | 16594 | 3802 | **75048** |
|  |  | ***Pixel (T10)*** | 13106 | 12418 | 4940 | 4472 | 5270 | 11724 | 4231 | 16295 | 4059 | **76515** |

**Table IVs.** AS positive droplets with each concentration in wet samples (T0) and dry residue (T10). Note that in some cases the number of segmented pixels is different between the wet sample and the dry residue. Concentrations C1 = 500 TU·μL^-1^, C2 = 800 TU·μL^-1^, C3 = 1000 TU·μL^-1^, C4 = 1500 TU·μL^-1^, C5 = 2000 TU·μL^-1^, C6 = 2500 TU·μL^-1^, C7 = 3000 TU·μL^-1^, C8 = 3500 TU·μL^-1^, C9 = 4000 TU·μL^-1^.

| **Method** | **Experimental Groups** | | **Number of negative droplets and pixels** | | | | | | | | | | |
| --- | --- | --- | --- | --- | --- | --- | --- | --- | --- | --- | --- | --- | --- |
|  |  |  | **PBS** | | | | | | | | | | |
|  |  |  | ***C0*** | ***C1*** | ***C2*** | ***C3*** | ***C4*** | ***C5*** | ***C6*** | ***C7*** | ***C8*** | ***C9*** | ***TOTAL*** |
| ***PLS-DA*** | ***Training*** | ***Droplet (T0/T10)*** | 1 | 2 | 2 | - | - | - | 2 | - | 2 | - | 9 |
|  |  | ***Pixels (T0)*** | 764 | 1605 | 1600 | - | - | - | 1061 | - | 1134 | - | 6164 |
|  |  | ***Pixels (T10)*** | 863 | 1400 | 1700 | - | - | - | 1705 | - | 1323 | - | 6991 |
|  | ***Test*** | ***Droplet (T0/T10)*** | - | - | - | 1 | 1 | 1 | - | 1 | - | 1 | 5 |
|  |  | ***Pixels (T0)*** | - | - | - | 1190 | 945 | 1418 | - | 950 | - | 1041 | 5544 |
|  |  | ***Pixels (T10)*** | - | - | - | 1575 | 1431 | 2001 | - | 1109 | - | 1476 | 7592 |
|  | **TOTAL** | ***Droplet (T0/T10)*** | 1 | 2 | 2 | 1 | 1 | 1 | 2 | 1 | 2 | 1 | 14 |
|  |  | ***Pixels (T0)*** | 764 | 1605 | 1600 | 1190 | 945 | 1418 | 1061 | 950 | 1134 | 1041 | **11708** |
|  |  | ***Pixels (T10)*** | 863 | 1400 | 1700 | 1575 | 1431 | 2001 | 1705 | 1109 | 13223 | 1476 | **14583** |
| ***FFNN*** | ***Training*** | ***Droplet (T0/T10)*** | 1 | 1 | 1 | - | - | - | 1 | - | 1 | - | 5 |
|  |  | ***Pixels (T0)*** | 764 | 1049 | 1051 |  |  |  | 666 |  | 768 |  | 4298 |
|  |  | ***Pixels (T10)*** | 863 | 810 | 1044 | - | - | - | 994 | - | 669 | - | 4380 |
|  | ***Validation*** | ***Droplet (T0/T10)*** | - | 1 | 1 | - | - | - | 1 | - | 1 | - | 4 |
|  |  | ***Pixels (T0)*** | - | 556 | 549 | - | - | - | 395 | - | 366 | - | 1866 |
|  |  | ***Pixels (T10)*** | - | 590 | 656 | - | - | - | 711 | - | 654 | - | 2611 |
|  | ***Test*** | ***Droplet (T0/T10)*** | - | - | - | 1 | 1 | 1 | - | 1 | - | 1 | 5 |
|  |  | ***Pixels (T0)*** | - | - | - | 1190 | 945 | 1418 | - | 950 | - | 1041 | 5544 |
|  |  | ***Pixels (T10)*** | - | - | - | 1575 | 1431 | 2001 | - | 1109 | - | 1476 | 7592 |
|  | **TOTAL** | ***Droplet (T0/T10)*** | 1 | 2 | 2 | 1 | 1 | 1 | 2 | 1 | 2 | 1 | 14 |
|  |  | ***Pixels (T0)*** | 764 | 1605 | 1600 | 1190 | 945 | 1418 | 1061 | 950 | 1134 | 1041 | **11708** |
|  |  | ***Pixels (T10)*** | 863 | 1400 | 1700 | 1575 | 1431 | 2001 | 1705 | 1109 | 13223 | 1476 | **14583** |

**Table Vs.** PBS negative controls droplets with each concentration in wet samples (T0) and dry residue (T10). Note that in some cases the number of segmented pixels is different between the wet sample and the dry residue. Initial stock titer C0 = 20·10^3^ TU·μL^-1^. Serial dilutions C1 = 500 TU·μL^-1^, C2 = 800 TU·μL^-1^, C3 = 1000 TU·μL^-1^, C4 = 1500 TU·μL^-1^, C5 = 2000 TU·μL^-1^, C6 = 2500 TU·μL^-1^, C7 = 3000 TU·μL^-1^, C8 = 3500 TU·μL^-1^, C9 = 4000 TU·μL^-1^.

| **Method** | **Experimental Groups** | | **Number of negative droplets and pixels** | | | | | | | | | |
| --- | --- | --- | --- | --- | --- | --- | --- | --- | --- | --- | --- | --- |
|  |  |  | **AS** | | | | | | | | | |
|  |  |  | ***C1*** | ***C2*** | ***C3*** | ***C4*** | ***C5*** | ***C6*** | ***C7*** | ***C8*** | ***C9*** | ***TOTAL*** |
| ***PLS-DA*** | ***Training*** | ***Droplet (T0/T10)*** | 3 | 3 | - | - | - | 3 | - | 3 | - | 12 |
|  |  | ***Pixel (T0)*** | 2642 | 2651 | - | - | - | 1847 | - | 2826 | - | 9966 |
|  |  | ***Pixel (T10)*** | 2691 | 2225 | - | - | - | 2282 | - | 2678 | - | 9876 |
|  | ***Test*** | ***Droplet (T0/T10)*** | - | - | 1 | 1 | 1 | - | 1 | - | 1 | 5 |
|  |  | ***Pixel (T0)*** | - | - | 1528 | 1254 | 1464 | - | 1711 | - | 1686 | 7643 |
|  |  | ***Pixel (T10)*** | - | - | 1338 | 1430 | 1756 | - | 1620 | - | 1890 | 8034 |
|  | **TOTAL** | ***Droplet (T0/T10)*** | 3 | 3 | 1 | 1 | 1 | 3 | 1 | 3 | 1 | 17 |
|  |  | ***Pixel (T0)*** | 2642 | 2651 | 1528 | 1254 | 1464 | 1847 | 1711 | 2826 | 1686 | 17609 |
|  |  | ***Pixel (T10)*** | 2691 | 2225 | 1338 | 1430 | 1756 | 2282 | 1620 | 2678 | 1890 | 17910 |
| ***FFNN*** | ***Training*** | ***Droplet (T0/T10)*** | 2 | 2 | - | - | - | 2 | - | 2 | - | 8 |
|  |  | ***Pixel (T0)*** | 1811 | 1888 | - | - | - | 1124 | - | 2009 | - | 6832 |
|  |  | ***Pixel (T10)*** | 1985 | 1553 | - | - | - | 1493 | - | 2072 | - | 7103 |
|  | ***Validation*** | ***Droplet (T0/T10)*** | 1 | 1 | - | - | - | 1 | - | 1 | - | 4 |
|  |  | ***Pixel (T0)*** | 831 | 763 | - | - | - | 723 | - | 817 | - | 3134 |
|  |  | ***Pixel (T10)*** | 706 | 672 | - | - | - | 789 | - | 606 | - | 2773 |
|  | ***Test*** | ***Droplet (T0/T10)*** | - | - | 1 | 1 | 1 | - | 1 | - | 1 | 5 |
|  |  | ***Pixel (T0)*** | - | - | 1528 | 1254 | 1464 | - | 1711 | - | 1686 | 7643 |
|  |  | ***Pixel (T10)*** | - | - | 1338 | 1430 | 1756 | - | 1620 | - | 1890 | 8034 |
|  | **TOTAL** | ***Droplet (T0/T10)*** | 3 | 3 | 1 | 1 | 1 | 3 | 1 | 3 | 1 | 17 |
|  |  | ***Pixel (T0)*** | 2642 | 2651 | 1528 | 1254 | 1464 | 1847 | 1711 | 2826 | 1686 | 17609 |
|  |  | ***Pixel (T10)*** | 2691 | 2225 | 1338 | 1430 | 1756 | 2282 | 1620 | 2678 | 1890 | 17910 |

**Table VIs.** AS negative controls droplets with each concentration in wet samples (T0) and dry residue (T10). Note that in some cases the number of segmented pixels is different between the wet sample and the dry residue. Concentrations C1 = 500 TU·μL^-1^, C2 = 800 TU·μL^-1^, C3 = 1000 TU·μL^-1^, C4 = 1500 TU·μL^-1^, C5 = 2000 TU·μL^-1^, C6 = 2500 TU·μL^-1^, C7 = 3000 TU·μL^-1^, C8 = 3500 TU·μL^-1^, C9 = 4000 TU·μL^-1^.

***Per-droplet classification using a droplet-averaged PLS-DA model***

Additionally, we re-analyzed the data using the mean reflectance spectra for each droplet within the same experimental groups used in the pixel-based PLS-DA method previously described. It is important to note that, in this case, individual PA spectra from all pixels within each droplet were averaged and then processed following a similar methodology. Briefly, the program used for this average-droplet analysis was Unscramble X version 10.4 (Camo Software AS, Oslo, Norway). The Savitzky-Golay filter applied for preprocessing of averaged spectra employed 5th-order polynomials within 32-point bands. Samples are labelled in two classes: positive droplets (with lentiviral particles) are “+1”, and negative droplets (controls, fluid without lentiviral particles) are “-1”. The classifier returns a continuous output variable. The resulting boxplots show predicted values for each droplet, and their uncertainty. A full (leave-one-out) cross-validation was then applied to generate the output for classification.

The obtained results are shown in Figure 7.

**Fig. 7 – Predicted per-droplet classification using the droplet-averaged PLS-DA model**. Red lines correspond to the classification value predicted for each droplet.

LP-i = droplet number “I”, positive sample (red box).

C-i = droplet number “I”, negative (control) sample (green box).

| **a**, liquid samples (T0) in PBS fluid.  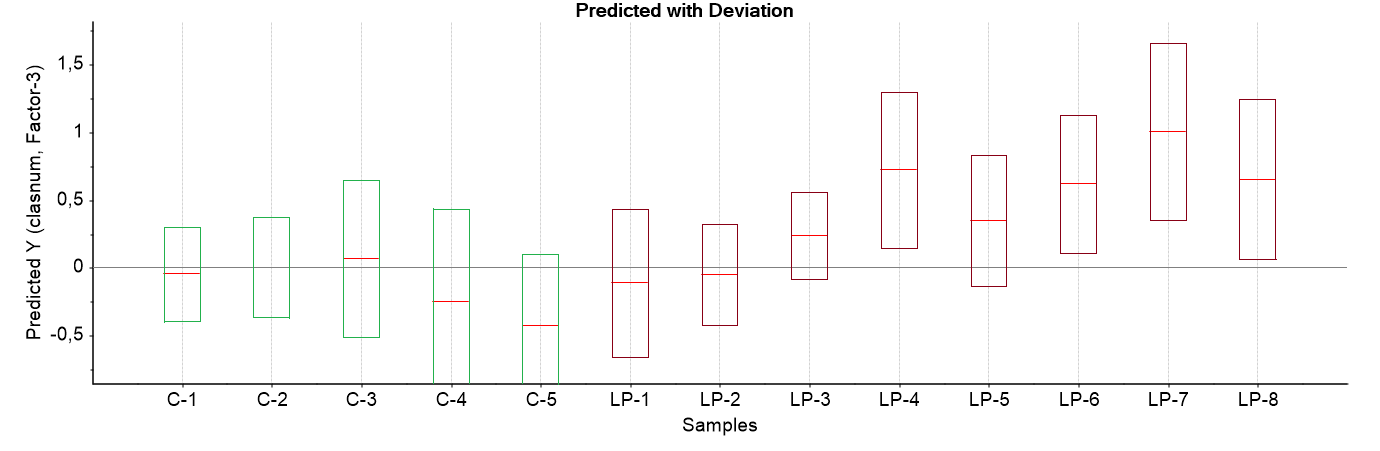 |
| --- |
| **b**, liquid samples (T0) in artificial saliva.  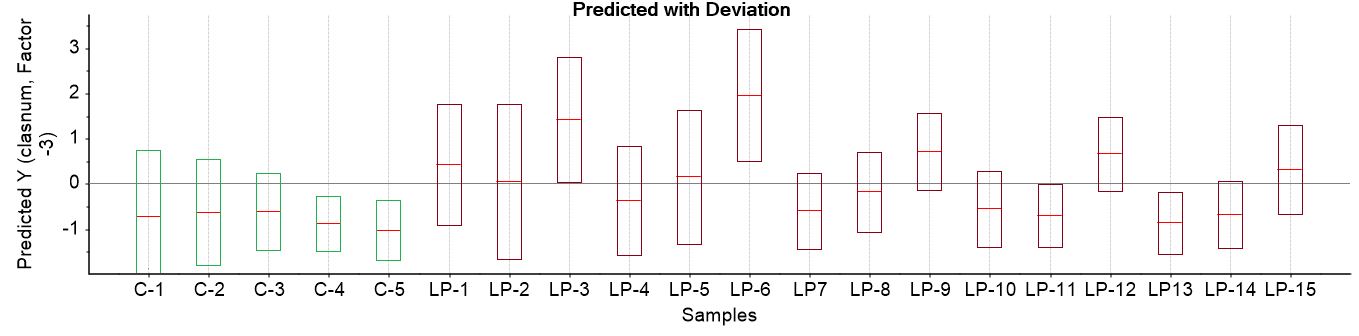 |
| **c**, dry residue (T10) in PBS fluid.  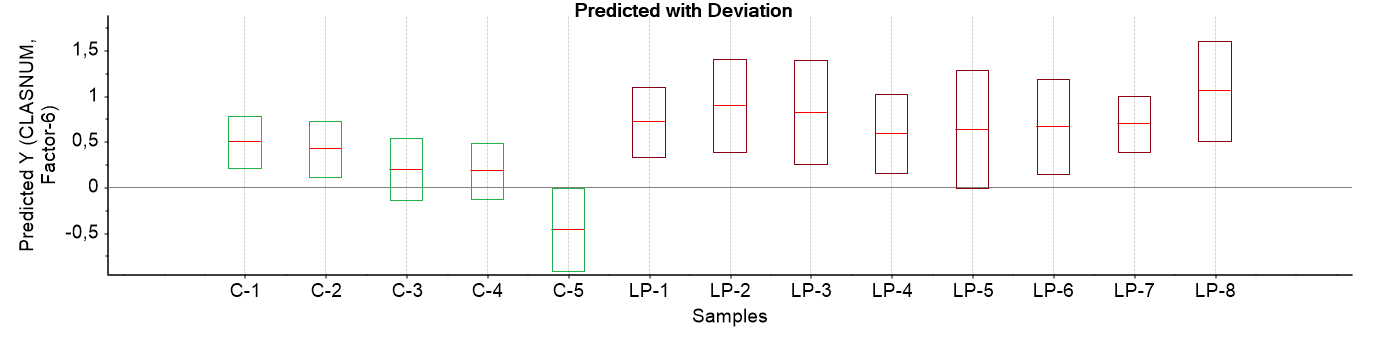 |
| **d**, dry residue (T10) in artificial saliva.  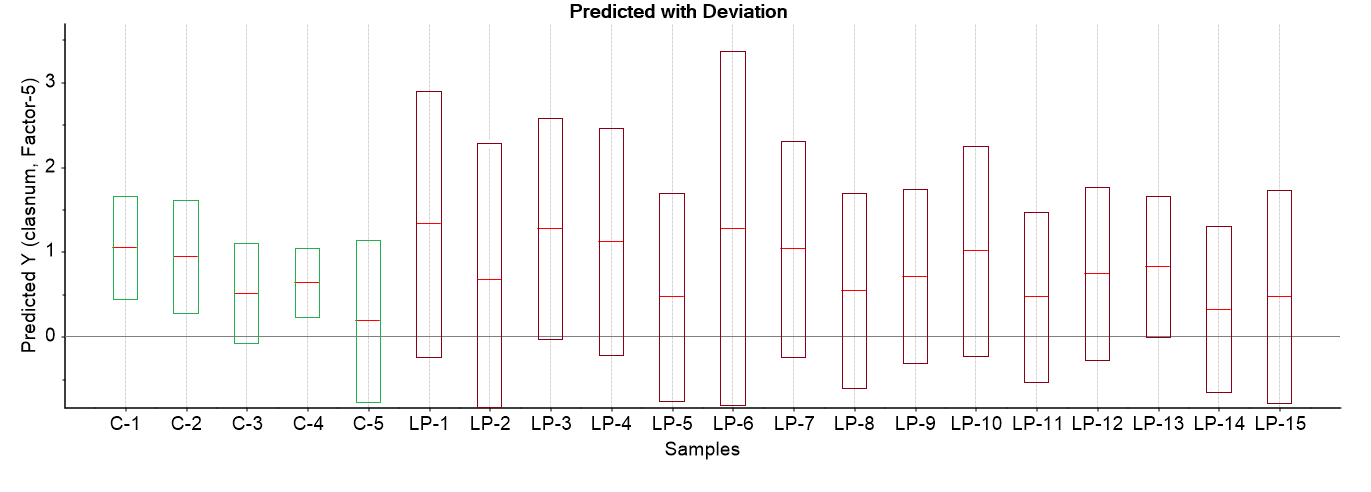 |
